# Self-medication practices and associated factors among pregnant women in Oromia, Ethiopia: institutional based cross-sectional study

**DOI:** 10.3389/fmed.2025.1489990

**Published:** 2025-03-26

**Authors:** Hadra Nuri Ahmed, Beyene Sisay Damtew, Wondu Abera Bezabih, Elias Bekele Wakwoya, Hinsermu Bayu Abdi, Getahun Tiruye

**Affiliations:** ^1^Nursing Department, Asella Teaching and Referral Hospital, Asella, Ethiopia; ^2^Department of Midwifery, College of Health Sciences, Arsi University, Asella, Ethiopia

**Keywords:** self-medication, pregnant woman, antenatal care, Asella town, Ethiopia

## Abstract

**Background:**

Self-medication, the practice of using medications without medical prescription, is a widespread phenomenon, particularly among pregnant women. This practice can lead to serious adverse effects on both the mother and the fetus, including drug interactions, birth defects, and premature labor. Despite its potential risks, the prevalence and factors associated with self-medication during pregnancy in Ethiopia remain understudied.

**Objective:**

This study aimed to assess the prevalence of self-medication practices and identify the factors associated with it among pregnant women attending antenatal care in Oromia, Ethiopia.

**Methods:**

A cross-sectional study was conducted from August to September 2023, involving 418 pregnant women attending antenatal care at public health institutions in Asella. A structured questionnaire given by an interviewer was used to gather data. Using SPSS version 25, the gathered data was cleaned, coded, and examined. Binary logistic regression analysis was used to determine the factors associated to self-medication, and descriptive statistics were used to summarize the data.

**Results:**

Overall the prevalence of self-medication practice among pregnant women was 39.5% (95%CI: 34.7–44.7%). Factors associated with self medication practice during pregnancy are; Primigravida women (AOR 2.18, 95% CI: 1.08–3.38), those with unintended pregnancies (AOR 1.65, 95% CI: 1.20–1.70), lacking health education on self-medication during (AOR 1.50, 95% CI: 1.45–2.55), those previous pregnancy and delivery related problem (AOR 1.6, 95% CI 1.55–2.65) were significantly associated with self-medication practice.

**Conclusion and recommendation:**

Self-medication is a prevalent practice among pregnant women in Asella, Ethiopia. To mitigate the risks associated with self-medication, it is crucial to implement comprehensive health education programs targeting pregnant women, particularly primigravida women and those with unintended pregnancies. These programs should emphasize the dangers of self-medication, the importance of seeking professional medical advice, and the proper use of medications during pregnancy.

## Background

Self-medication, according to the World Health Organization (WHO), is using medications to treat self-diagnosed conditions without consulting a healthcare professional. This definition includes the use of both modern medicines and herbal remedies. For pregnant women, self-medication specifically refers to the practice of using medications to treat self-diagnosed health issues without seeking guidance from a healthcare professional (1). Self-medication encompasses a range of practices, including using over-the-counter medications, inconsistently taking prescribed drugs, utilizing leftover medications, and consuming both conventional and herbal remedies without professional medical guidance (2, 3).

In developing countries like Ethiopia, where healthcare access is limited and resources are scarce, self-medication is a growing trend. This is particularly concerning during pregnancy, as physiological and anatomical changes make women susceptible to various symptoms like headaches, nausea, vomiting, and edema, potentially leading them to self-medicate (4). Due to these common pregnancy symptoms, many women resort to self-medication (5).

Pregnant women are often involved in self-medication due to herbal medicines are more accessible, affordable, and readily available. They may assume that natural treatments have no problem (6).

While the specific self-medication practices, frequency, and motivations vary across regions, the issue poses a significant global public health threat during pregnancy, demanding attention due to the potential harm to both maternal and fetal health (7). Studies indicate that self-medication is a widespread issue among pregnant women, with global prevalence estimates ranging from 22 to 44% (8). Study from Africa shows that practice of self medication during pregnancy range from 32 to 43.5% (8–10). However study done in Ethiopia shows that 15.5% (11). A systematic review of 33 studies, primarily from Iran, revealed that herbal medicine use during pregnancy is prevalent, though with a wide range of reported usage, from 19.2 to 90.2%. Women frequently used these remedies to alleviate pregnancy-related pain, often based on recommendations from their social networks (12). A study conducted in Iran found that 32% of pregnant women engaged in self-medication (8). In resource-limited settings, particularly in Africa, self-medication during pregnancy has been reported at significantly higher rates, reaching up to 85% (7, 13, 14). Even in areas with more resources, certain groups are more prone to self-medication during pregnancy: women with formal education, those who are unemployed, women with low socioeconomic status, and those in early pregnancy (15).

In Africa, self-medication during pregnancy is a common practice, involving both conventional and herbal remedies, with notably high prevalence rates: 72.4% in Nigeria, 59.9% in the Democratic Republic of Congo, and 46.24% in Tanzania (16–18). A study in Hossana Town, Southern Ethiopia, revealed a high prevalence of herbal medicine use during pregnancy, with 73.1% of women reporting its use in their current pregnancy (19).

Self-medicating while pregnant poses a significant risk to both the mother’s and the baby’s well-being (20) Self-medication during pregnancy can lead to a range of harmful effects on the fetus, including structural abnormalities, functional impairments, toxicity, birth defects, and other potential developmental issue (21). Drug exposure during pregnancy is a significant contributor to birth defects, accounting for over 10% of cases. In addition to the risks of birth defects and developmental issues, self-medication during pregnancy can also lead to premature birth, low birth weight, feeding difficulties later in the child’s life, and respiratory problems (22).

Pregnant women, due to their unique physiological state, are particularly vulnerable and often engage in frequent and repeated self-medication, driven by the desire to prevent miscarriage and manage pregnancy-related health issues (23). While pregnant women often turn to herbal remedies, this practice is concerning because the unknown composition and safety of these products mean they could contain substances that induce abortion, premature birth, uterine contractions, or fetal harm (24).

A systematic review examining herbal medicine consumption across 13 African nations revealed a wide range of usage. South Africa reported the highest prevalence, with 33 to 93% of the population using herbal remedies. Nigeria also demonstrated significant usage, ranging from 31.4 to 68%. Mali showed a high rate of 86%, while Zimbabwe’s consumption fell between 52 and 69%. Tanzania reported 55% usage. Several countries, including Ethiopia (48.6–50.4%), Ghana (under 50%), and Côte d’Ivoire (35%), exhibited moderate usage. Lower levels of herbal medicine consumption were observed in Malawi (25.7%), Kenya (12%), Zambia (21%), and Uganda (20%). This review highlights the diverse patterns of herbal medicine use across the African continent (25).

Different countries have different reasons why pregnant women in those countries choose to self-medicate. It is linked to variables like gestational age, occupation, income, age, access to medications, perception of the risk of self-medication, influence from friends or relatives regarding past medication use, and long wait times in medical facilities (21, 26–28).

While Ethiopia has achieved significant reductions in maternal and child mortality over the past decade, progress in lowering neonatal mortality has been slower and has stagnated in recent years (29). In Ethiopia, self-medication during pregnancy plays a key role in causing premature births and low birth weights, which are the main reasons babies die shortly after birth (29). Effectively addressing self-medication during pregnancy and delivery is crucial for safeguarding the health of both mother and child, and it directly contributes to achieving Sustainable Development Goal (SDG) 3.2, which focuses on reducing neonatal mortality (30).

Despite the known dangers of self-medication during pregnancy in Ethiopia, there’s a lack of research on how widespread it is and what factors contribute to it. This study aims to provide crucial data to inform policymakers and stakeholders, enabling them to create strategies and interventions to mitigate the risks associated with self-medication in pregnant women.

## Materials and methods

### Study area

This study was conducted in Asella public health institutions, located at the South-East Ethiopia. The town is found in Oromia regional state, Arsi zone 175 km away from the capital city Addis Ababa. Currently, the total population of the town is estimated to be 139, 537 out of which 69, 459 are males and 70,078 are females. There are two government health centers, one teaching Hospital, two private Hospitals, three Non-governmental organization (NGO) clinics, 18 medium private clinics, and 27 pharmacy and drugs shops. Annually, Pregnant women who receive ANC follow-up at Asella Public Health Institution were estimated to be 5,176.

### Study design and period

This study used a cross-sectional design. It was conducted from August 1 to September 30, 2023, in healthcare institutions.

### Population, inclusion, and exclusion criteria

Source population: All pregnant women receiving antenatal care (ANC) services at public health facilities in Asella.

Study population: The study included pregnant women who were randomly selected from those attending prenatal checkups at Asella’s public health clinics during the data collection period.

Inclusion criteria: The study included all pregnant women who received prenatal care at Asella’s public health clinics within the study’s timeframe.

Exclusion criteria: Pregnant women with severe illnesses and those who refused to participate were excluded from the study.

### Sample size and sampling method

The study’s sample size was calculated using a formula for single population proportions, using a self-medication prevalence rate of 44.8% observed in pregnant women in Gondar (31) 95% confidence level, 5% marginal error, and 10% non-response rate. Using the formula


$$n=\frac{{\left(Z_{\frac{\propto }{2}}\right)}^{2}P\left(1-P\right)}{E^{2}}$$

Where;

n = was the sample size.

Z = was the reliability coefficient associated with 95% confidence level 1.96,

p = prevalence of self-medication from previous data, taken to be 44.8%.

E = Margin of error or maximum acceptable difference = 0.05.

$n=\frac{{\left(1.96\right)}^{2}\left(0.448\right)\left(0.552\right)}{{\left(0.05\right)}^{2}}$ = 380 + 10% = 418.

n = 380 + 10% non-respondent rate.

Therefore, the total sample size was 418 pregnant women.

### Sampling procedure

**Figure 1:**
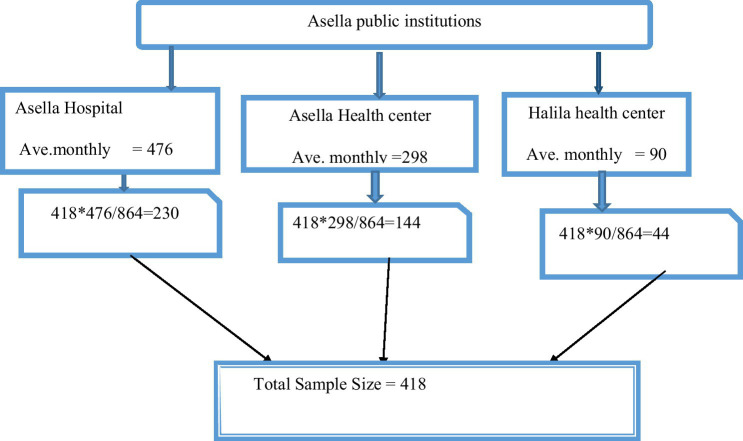
Schematic representation of sampling techniques on self-medication practices and associated factors in Asella public health institutions, 2023.

This study used systematic random sampling to select participants from three public health facilities in Asella offering antenatal care (ANC): a hospital and two health centers. The total number of pregnant women attending ANC at these facilities was estimated based on the previous two months’ records. The final sample size of 418 was proportionally distributed among the facilities according to their average monthly ANC patient volume. To determine the sampling interval, the researchers used the previous year’s data for the same period, which showed 864 women attended ANC. Dividing this total by the desired sample size (864/418) resulted in a sampling interval of 2. Therefore, every second pregnant woman arriving for ANC at each facility was selected for the study until the allocated sample size for that facility was reached. ‘N’ represents the total number of pregnant women attending ANC at each facility, and ‘Kth’ represents the sampling interval (Figure 1).

### Dependent variable

Self-medication practice.

### Independent variables

Socio-demographic characteristics: age, marital status, ethnicity, religion, occupation, monthly income, educational status, husband’s educational status, and place of residence.

Obstetric-related factors: Gestational age, gravidity, number of children, health problem during pregnancy and history of abortion.

Health service delivery-related factors: Lack health education, Health insurance, Baying medication without prescription and long waiting time.

### Operational definition

Self-medication practice: Pregnant women engaging in self-medication are those who, on their own initiative, use medications to treat self-diagnosed illnesses or symptoms during their current pregnancy (32).

Conventional medicines: These are medications obtained from established medical practitioners like physicians, nurses, and other recognized healthcare providers (33).

Herbal medicines: is the practice of using plants with medicinal properties to prevent and treat illnesses (34).

### Data collection tool

Data was collected through face-to-face interviews using a structured questionnaire. This questionnaire, originally in English, was translated and back-translated to Afan Oromo to maintain consistency. Adapted from various sources, it covered all variables relevant to the study objectives. Four BSc Midwives conducted the interviews from August 1 to September 30 (11, 35, 36). This study received ethical approval from the Arsi University Ethical Review Board. Before participation, all women were fully informed about the study’s purpose, procedures, potential risks, and benefits. They were assured of confidentiality and anonymity, and their informed consent was obtained. Women who declined to participate were excluded. The questionnaire used for data collection consisted of three sections: socio-demographic information, health service-related issues, and self-medication practices during pregnancy.

### Data quality control

Before the main study, the questionnaire was pretested at Eteya Health Center with 21 women (5% of the intended sample). Based on the pretest findings, the questionnaire was revised to improve clarity and include additional questions, as well as to reorder some questions. The data collectors received 2 days of training on the study’s objectives, data collection procedures, questionnaire details, interviewing techniques, and ensuring confidentiality. To maintain data quality, completed questionnaires were reviewed daily for completeness and relevance.

### Ethical consideration

This study received ethical approval from the Arsi University Institutional Review Board (reference number AU/H/S/C/120/244/15). Permission was also granted by the Asella City Administration Health Bureau and the directors of all participating health facilities. Before data collection, data collectors explained the study’s purpose, methods, benefits, and risks to each pregnant woman. Verbal consent was obtained after confirming their willingness to participate. Throughout the study, including data collection, analysis, and dissemination of results, participant privacy and confidentiality were strictly maintained.

### Statistical analysis

Data was manually reviewed for completeness, coded, and entered into Epi Data 3.1 software before being exported to SPSS 25 for analysis. Descriptive statistics, including frequencies, means, standard deviations, and percentages, were calculated to summarize socio-demographic and other relevant variables. Results were presented in tables, graphs, and figures. Logistic regression was used to identify factors associated with self-medication during pregnancy. The Hosmer-Lemeshow test assessed the model’s fit. Variables with a *p*-value less than 0.25 in the Bivariate analysis were included in the multivariable logistic regression. Statistical significance was defined as a *p*-value less than 0.05. Adjusted odds ratios and 95% confidence intervals were used to report the strength of associations, with statistical significance declared at *p* < 0.05.

## Results

### Socio-demographic, economic and health-related characteristics

The study included 418 pregnant women with a 100% participation rate. The majority of participants were between 23 and 27 years old, with an average age of 26.59 years (sd ± 5.15). A large majority (96.9%, 405 women) were married. Regarding religious affiliation, 46.9% (196 women) were Muslim. Ethnically, 60.3% (252 women) identified as Oromo (Table 1).

**Table 1:** Socio-demographic and economic characteristics of pregnant women in Asella public health institution, Ethiopia, 2023.

Variables	Category	Frequency (*n* = 418)	Percentage (%)
Age of respondents in years	18–22	97	23.2
	23–27	146	34.9
	28–32	119	28.9
	33–35	32	7.7
	>36	24	5.7
Marital status	Married	405	96.9
	Divorced	2	0.5
	Widowed	2	0.5
	Single	9	2.2
Religion	Orthodox	165	39.5
	Muslim	196	46.9
	Protestant	52	12.4
	Catholic	5	1.2
Ethnicity	Amhara	116	27.8
	Oromo	252	60.3
	Tigre	15	3.6
	Gurage	23	5.5
	Other	12	2.9
Educational status of women	No formal education	58	13.9
	Primary education	177	42.3
	Secondary education	96	23.0
	College and above	87	20.8
Educational status of husband	No formal education	38	9.0
	Primary education	138	32.9
	Secondary education	112	26.7
	College and above	117	27.9
Occupation	Housewife	158	37.8
	Government employee	71	17.0
	Private employee	77	18.4
	Merchant	11	2.6
	daily laborer	13	3.1
	Farmer	72	17.2
	Student	16	3.8
Place of residence	Urban	232	55.5
	Rural	186	44.5
Average monthly income	≤1,025	60	14.4
	1,026–3,995	267	63.9
	>3,996	91	21.8

### Obstetric characteristics of the respondents

Of the 418 pregnant participants, 39.2% were in their third trimester (7–9 months), while 24.9% were in their first trimester (1–3 months). Regarding their pregnancy history, 45.5% were first-time mothers (primigravida), 34.7% had had multiple pregnancies (multigravida), and 19.9% had had many pregnancies (grand multipara) (Table 2).

**Table 2:** Obstetric characteristics of pregnant women in Asella public health institutions, Ethiopia, 2023.

Variable	Category	Frequency	Percent
Gravida	Primigravida	190	45.5
	Multi gravida	145	34.7
	Grand para	83	19.9
Number of children	No child	190	45.5
	1–3	124	29.9
	>3	104	24.6
History of abortion	Yes	89	21.3
	No	329	78.7
Un intended pregnancy	Yes	181	43.3
	No	237	56.7
Gestational age	First Trimester	104	24.9
	Second Trimester	150	35.9
	Third Trimester	164	39.2
Not received health education on the risk of self-medication during pregnancy at ANC visit	Yes	147	35.2
	No	271	64.8
History of health problems during the current pregnancy	Yes	128	30.6
	No	290	69.4
Previous pregnancy and delivery-related problems	Yes	96	23
	No	322	77

### Self-medication practice during pregnancy

The study showed that while a majority (60.5%) of individuals did not engage in self-medication, a significant portion (39.5%) did. Among those who self-medicated, the most common practice was using modern medicine alone (23.3%). Traditional medicine was used by a smaller percentage (10.0%), and a combination of modern and traditional medicine was used by 6.2%. The most common sources of medication were private clinics (8.4%) and pharmacies (17.7%), followed by less formal options like home/family, friends, and markets.

**Figure 2:**
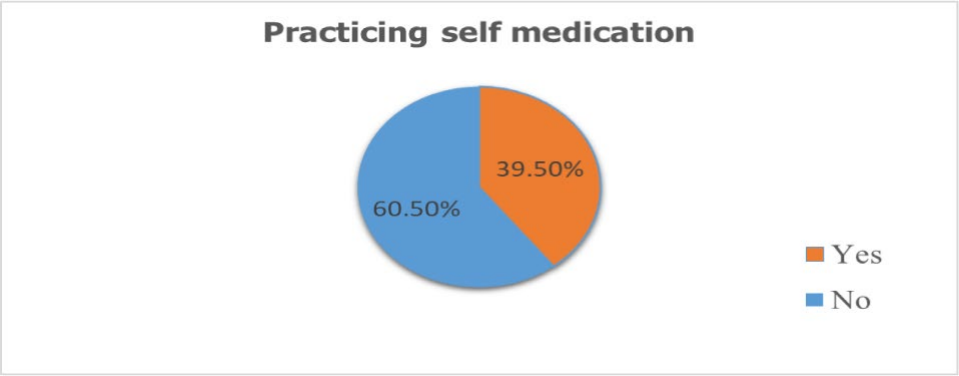
Magnitude of self-medication among pregnant mothers attending antenatal care at Asella public health institutions Arsi one, Oromia, Ethiopia, 2023.

This study found that 39.5% of pregnant women engaged in self-medication (Figure 2).

### Reason for self-medication

**Figure 3:**
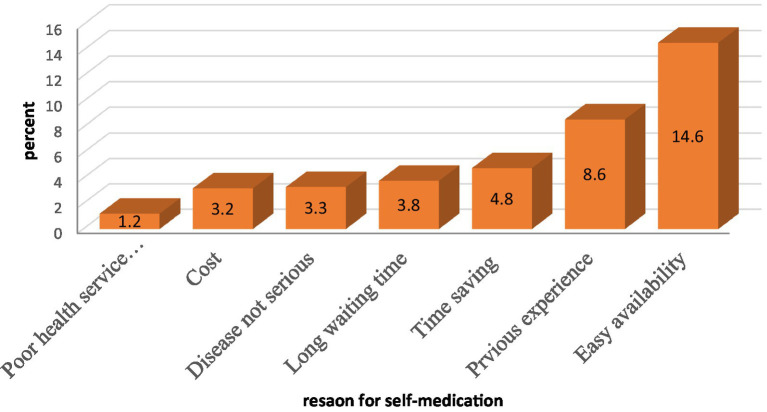
Reason for self-medication of the respondent of pregnant women in Asella town, Ethiopia, 2023.

The primary reason pregnant women reported for self-medicating was the easy accessibility of medications (14.6%). Other contributing factors included saving time (4.8%), underestimating the severity of their illness (3.3%), cost considerations (3.2%), and prior successful self-medication (3.8%). Less frequent reasons were dissatisfaction with health services (1.2%) and lengthy wait times (3.8%) (Figure 3).

### Major complaints after self-medication

**Figure 4:**
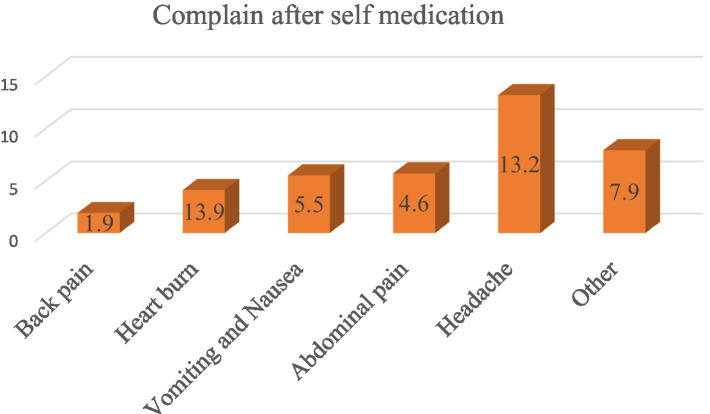
Major complaints after self-medication of the respondent of pregnant women in Asella Town, Ethiopia, 2023.

One hundred sixty five [165 (39.5%)] of pregnant women experienced health problems related to complaints after self-medication practices. Of these, headache 58 (13.9), abdominal pain 28 (5.7), Nausea and vomiting 25 (5.5) heartburn 17 (4.6), back pain 8 (1.9), and others 29 (7.9) were some of the common health problems encountered by pregnant women after practicing self medications (Figure 4).

### History of Ethiopian traditionalist medicine and herbs use

**Figure 5:**
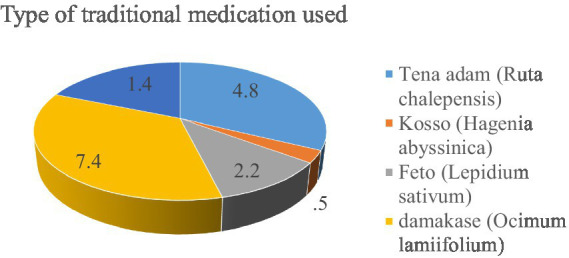
History of traditional medicine and types of herbs used by the participants in Asella Town, Ethiopia.

The study showed that the most commonly used traditional medication was Tena Adam (*Ruta chalepensis*) (4.8%), followed by Kosso (*Hagenia abyssinica*) (7.4%) and Feto (*Lepidium sativum*) (2.2%). A smaller percentage of respondents used Damakase (*Ocimum lamiifolium*) (0.5%) or other traditional medications (1.4%) (Figure 5).

### Factors associated with self-medication practice

The final model revealed that primigravidity, unplanned pregnancy, health education on self-medication, and prior pregnancy complications were significant predictors of self-medication. Primigravidas were 2.18 times more likely to self-medicate than grand multiparas (AOR = 2.18, 95% CI: 1.18, 3.38). Women with unplanned pregnancies were 1.65 times more likely to self-medicate than those with planned pregnancies (AOR = 1.65, 95% CI: 1.60, 2.70). Additionally, women who did not receive health education on self-medication risks were 1.50 times more likely to practice self-medication (AOR = 1.50, 95% CI: 1.45, 2.55). Finally, women with a history of pregnancy and delivery complications were 1.60 times more likely to self-medicate (AOR = 1.60, 95% CI: 1.55, 2.65) (Table 3).

**Table 3:** Bivariate and multivariate analysis of factors associated with practicing self-medication in Asella town, Ethiopia, 2023 (*n* = 418).

Variables	Category	Self-medication		COR (95% C.I)	AOR (95% C.I.)	*P* (<0.05)
		Yes	No			
Educational status of women	No formal education	35 (60.3)	23 (39.7)	0.87 (0.51,1.48)	0.93 (0.52,1.66)	
	Primary education	71 (40.1)	106 (59.9)	0.38 (0.19,0.76)	0.54 (0.25,1.17)	
	Secondary education	27 (28.1)	69 (71.9)	1.49 (0.80,2.77)	1.52 (0.78,2.99)	
	College and above	32 (36.8)	55 (63.2)	1	1	
Gravid	Primi	99 (52.1)	91 (47.9)	1.40 (1.23,1.69)	2.18 (1.08,3.38)	0.01**
	Multi	41 (28.3)	104 (71.7)	1.01 (0.60,1.98)	1.61 (0.56,1.97)	
	Grand	25 (30.1)	58 (68.9)		1	
Unintended pregnancy	Yes	90 (49.7)	91 (50.3)	2.14 (1.43,3.19)	1.65 (1.60,2.70)	0.01**
	No	75 (31.6)	162 (68.4)	1	1	
Lack of family health insurance	Yes	74 (43.5)	96 (56.5)	1.33 (1.28,1.38)	1.83 (0.81,2.20)	
	No	91 (36.7)	157 (63.3)	1	1	
Not receiving health education at ANC visit	Yes	78 (53.1)	69 (46.9)	2.37 (1.56,3.61)	1.50 (1.45, 2.55)	0.01**
	No	87 (32.1)	184 (67.9)	1	1	
Current experience of any health problem	Yes	56 (43.8)	72 (68.9)	1.23 (0.85,1.97)	1.11 (0.93,2.46)	
	No	109 (37.6%)	181 (62.0)	1	1	
Previous pregnancy and delivery related problems	Yes	55 (57.3)	41 (42.7)	2.59 (1.62,4.11)	1.60 (1.55,2.65)	0.01**
	No	110 (34.2)	212 (65.8)	1		

‘1’ = reference, ** = *p* < 0.01, * = *P* < 0.05.

## Discussion

This study investigated self-medication practices during pregnancy in Asella town, Ethiopia, finding revealed that a prevalence of 39.5% (95%CI: 34.7–44.3%). These results are consistent with studies conducted in the United Arab Emirates (40%) and Tigray, Ethiopia (40.8%) (10, 37).

The finding of this study (39.5%) was higher than that reported in studies conducted in Rajshahi city, Bangladesh (12.2%), Jordan (32.4%), Nigeria (22.3%), Gulu district of Uganda (20%), central Ethiopia (26.6%), southeast Ethiopia (15.5%), and southwest Ethiopia (20.1%) (11, 14, 28, 38–41) respectively. Variations in the study setting, such as urban versus rural environments, or differences in the study period, may have played a role. Additionally, socio-cultural factors unique to Asella town, Ethiopia, might influence pregnant women’s healthcare practices and their reliance on self-medication.

However, the finding of this study (39.5%) was considerably lower than a finding reported from, Pakistan (76%), Iran (83%), Assiut City, Egypt (53.3%), Hiwot Fana Specialized Hospital (69.4%), and Gonder Hospital (44.8%) [16, 34, 35, 43, 45 (42)] respectively. The discrepancies in self-medication prevalence between this study (39.5%) and other studies (ranging from 44.8 to 83%) could be attributed to several factors. The study’s findings may be influenced by differences in study locations, participant numbers, and the social backgrounds of the women involved. Furthermore, varying drug regulations across countries could affect medication availability and cost, which in turn impacts self-medication practices.

Primigravidas were more self-medicate than grand multiparas, with an AOR: 2.18 (95%CI: 1.08, 3.38). This finding aligns with similar studies conducted in Iran, Uganda, and Bahir Dar, Ethiopia (21, 40, 43). This is because, Primigravidas may be more susceptible to self-medication due to their limited knowledge of pregnancy-related medication risks and the potential dangers to both the mother and fetus. This lack of awareness could make primigravidas vulnerable to harmful self-medication practices.

Women with unplanned pregnancies were significantly more likely to engage in self-medication than those with planned pregnancies, with an AOR: 1.65 (95% CI: 1.60, 2.70). This finding is consistent with research conducted in Tanzania (44) and Nigeria (45). The higher likelihood of self-medication among women with unplanned pregnancies (AOR 1.65) could be due to two factors. First, they may be less aware of the risks associated with self-medication during pregnancy. Second, unplanned pregnancies may lead to less health-seeking behavior, causing women to avoid seeking medical attention for their ailments and resort to self-medication instead.

Women who did not receive health education about the risks of self-medication during antenatal care visits were 1.50 times more likely to engage in self-medication practices compared to those who did (AOR = 1.50, 95% CI: 1.45, 2.55). This finding aligns with studies conducted in Addis Ababa, (28) in Harar town, and central Ethiopia (46).

Women who did not receive this education were more likely to self-medicate, potentially due to a lack of awareness about the potential negative effects on their health and the developing fetus. This suggests that comprehensive health education during ANC visits, addressing the risks of self-medication in pregnancy, could be a key strategy to reduce this practice among pregnant women.

Women with a history of pregnancy and delivery complications were 1.6 times more likely to engage in self-medication than those without such complications (AOR = 1.6, 95% CI: 1.55, 2.65). These findings are consistent with studies conducted in Addis Ababa, Ethiopia (28). First, these women may have prior experience using medications to treat pregnancy-related problems, leading them to self-medicate again for similar issues. Second, they might perceive these medications as safe based on their past experiences, despite potential risks during a new pregnancy.

### Strength and limitations of the study

This study’s strength is its inclusion of both herbal and conventional medicines in its investigation of self-medication practices among pregnant women. However, the study is limited by potential recall bias, as some respondents may have struggled to accurately remember the details of their self-medication practices.

Two potential limitations should be considered. Due to their first pregnancy, primigravida women might not have had prior exposure to certain medications, potentially skewing results regarding regular prenatal drug use. Additionally, participants might have underreported drug use or given inaccurate drug names due to social desirability bias.

## Conclusion

Self-medication during pregnancy is a significant concern, with a prevalence rate of 39.5%. Several factors contribute to this high prevalence, including being a first-time mother, having an unintended pregnancy, not receiving adequate health education during antenatal care, and having experienced previous pregnancy or delivery-related problems. These factors highlight the need for increased awareness and education about the risks of self-medication during pregnancy, as well as improved access to quality antenatal care.

## Data Availability

The raw data supporting the conclusions of this article will be made available by the authors, without undue reservation.
